# Supplementary material for: A deep learning–based score to evaluate multiple sequence alignments
Source: Mol Biol Evol. 2026 Jul 22;43(8):msag173. doi: 10.1093/molbev/msag173 (PMC13428293; doi:10.1093/molbev/msag173)
Supplement: msag173_Supplementary_Data [file msag173_supplementary_data.zip › revised_SOP_sup_mateial_1.02.docx]

A deep-learning-based score to evaluate multiple sequence alignments

**Supplementary Material**

Nimrod Serok^1,*^, Ksenia Polonsky^1,*^, Haim Ashkenazy^2^, Itay Mayrose^3^, Jeffrey L. Thorne^4,5^, Tal Pupko^1†^

^1^ [The Shmunis School of Biomedicine and Cancer Research](https://en-lifesci.tau.ac.il/lp-en-mcbb), George S. Wise Faculty of Life Sciences, Tel Aviv University, Tel Aviv 69978, Israel.

^2^ Department of Molecular Biology, Max Planck Institute for Biology Tübingen, Tübingen, Germany.

^3^ The School of Plant Sciences and Food Security, George S. Wise Faculty of Life Sciences, Tel Aviv University, Tel Aviv 69978, Israel

^4^ Department of Biological Sciences, North Carolina State University, Raleigh, NC 27695, USA.

^5^ Department of Statistics, North Carolina State University, Raleigh, NC 27695, USA.

^†^To whom correspondence should be addressed. Email: [talp@tauex.tau.ac.il](mailto:talp@tauex.tau.ac.il)

*These two authors equally contributed to this work

**Supplemental Table S1**. Hyperparameter and architectural specifications for the four trained models. The table summarizes all model configurations used in this study.

|  | Simulated | | Empirical | |  |
| --- | --- | --- | --- | --- | --- |
|  | *Model 1* | *Model 2* | *Model 1* | *Model 2* | Ref and remarks |
| Number of layers | 4 | 4 | 4 | 3 |  |
| Number of neurons in each layer | 256, 16, 128, 64 | 64, 128, 64, 512 | 64, 64, 512, 128 | 190, 180, 256 |  |
| Activation function | PReLU | | | | (He et al. 2015) |
| Output layer | Linear | | | |  |
| Normalization | Batch normalization | | | | (Ioffe and Szegedy 2015) |
| Dropout rate | 0.34 | 0.24 | 0.10 | 0.32 |  |
| Regularization | L2, strength parameter set to $6.77\times{10}^{-5}$ | L2,  strength parameters set to 1$.65\times{10}^{-5}$ | L1-L2 Elastic Net  strength parameters set to $2.77\times{10}^{-6}$ and $1.03\times{10}^{-5}$ | L1-L2 Elastic Net  strength parameters set to $2.83\times{10}^{-5}$ and 4$.16\times{10}^{-7}$ | (Zou and Hastie 2005) |
| Optimizer | ADAM | | | | (Kingma and Ba 2015) |
| Initial learning rate | 0.0001 | 0.0022 | $0.00009$ | 0.0022 |  |
| Callbacks | Learning rate scheduler, early stopping | | | |  |
| Mini-batch size | 128 | 32 | 64 | 64 |  |
| Maximum number of training epochs | 50 | | | |  |
| $\tau_{1}, \hat{n}$ | N/A | 1.33, 8 | N/A | 0.28, 8 |  |

**Supplemental Table S2. Effect of sequence divergence on the ability of *Model 2* and SoP to identify accurate alternative MSAs.** Three simulated datasets (IDs 112755, 10659, and 22902) were analyzed after scaling all branch lengths in the underlying phylogenetic tree by factors of 1, 2, 4, 8, or 16. For each branch-length scaling factor, sequences were simulated, realigned, and 400 alternative MAFFT-based MSAs were generated. Alternative MSAs were ranked according to their true alignment accuracy, based on the the $d_{seq}$ measure. The table reports the rank of the MSA selected by either *Model 2* or the SoP scoring scheme. Lower ranks (in bold) indicate selection of a more accurate alignment.

|  | Model 2 | | | SoP | | |
| --- | --- | --- | --- | --- | --- | --- |
| BL factor | 112755 | 10659 | 22902 | 112755 | 10659 | 22902 |
| 1 | 18 | **5** | 55 | 18 | 71 | **7** |
| 2 | 14 | **1** | **1** | **2** | 13 | 40 |
| 4 | 3 | 1 | 1 | 3 | 1 | 1 |
| 8 | 1 | **1** | 1 | 1 | 296 | 1 |
| 16 | 1 | **71** | 339 | 1 | 400 | **312** |

**Supplemental
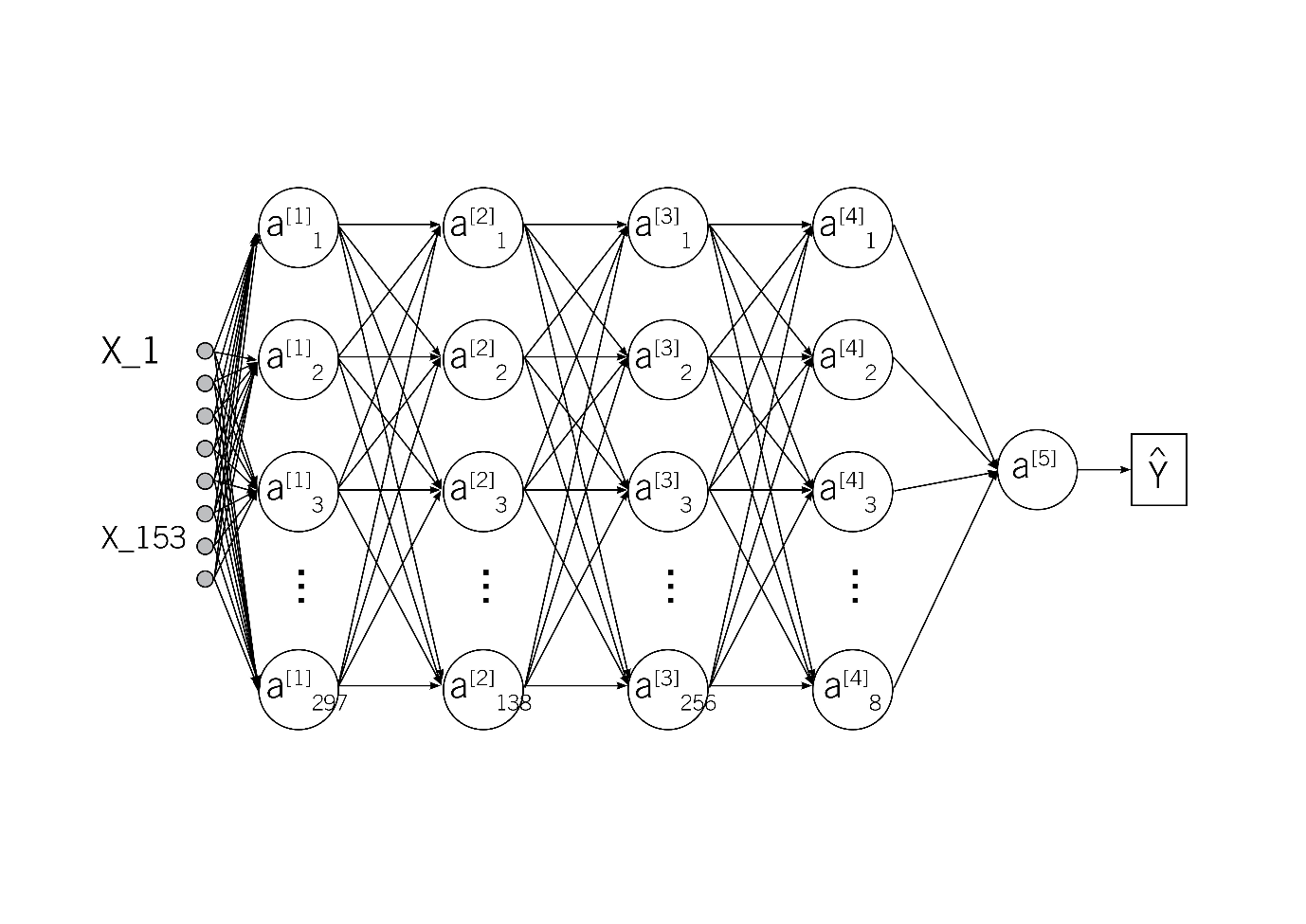
Figure S1**. A fully connected deep network architecture for *Model 1*. The number of nodes in the hidden layer equals the number of features (153 when analyzing simulated MSAs). The last layer is a sigmoid activation function that provides values between zero and one, predicting the distance between two MSAs according to $d_{seq}$. The number of layers, nodes in each layer and additional hyperparameters were optimized based on the validation dataset.


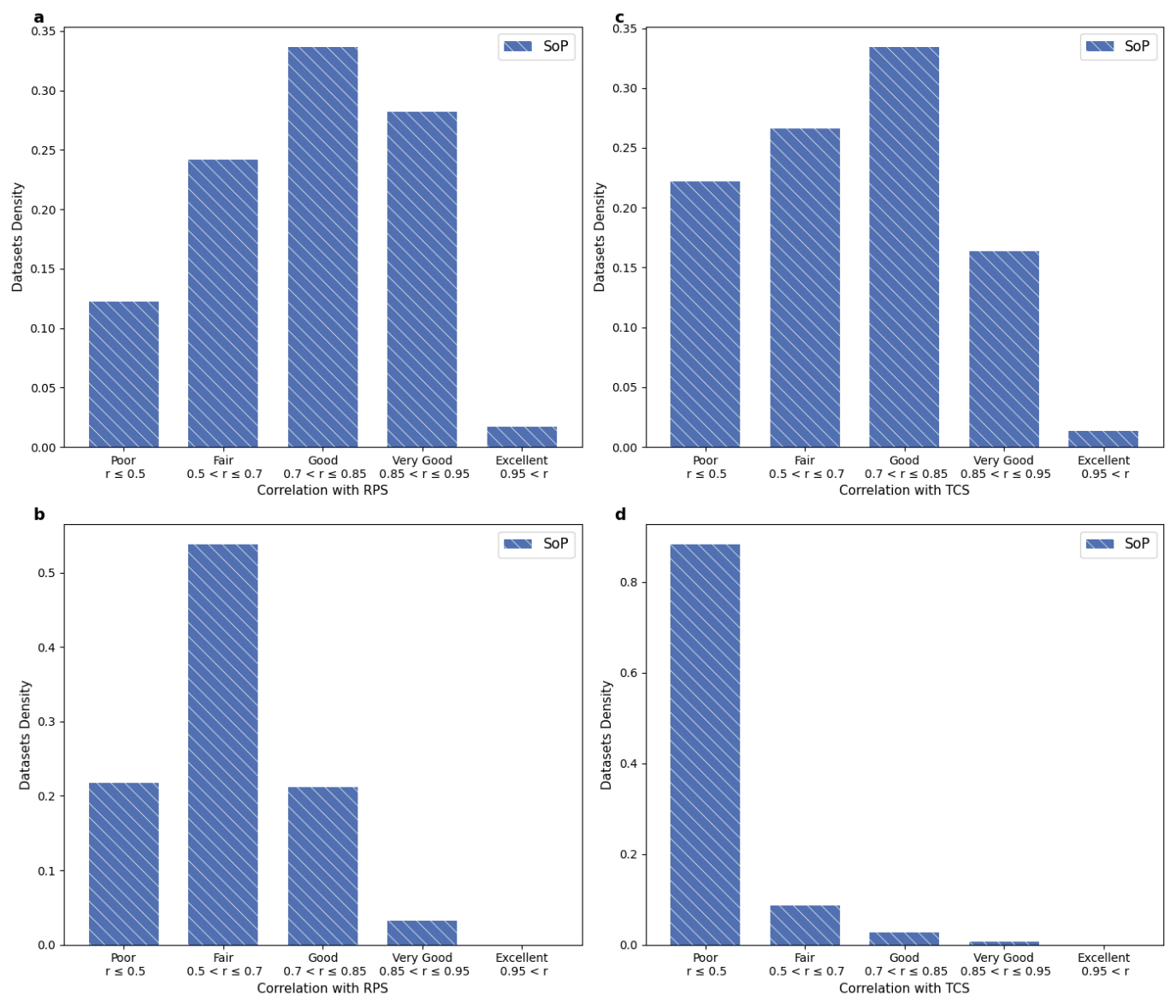


**Supplemental Figure S2**. (a) Distribution of Pearson correlation coefficients of SoP metric and RPS metric (from reference MSA) across 294 empirical BAliBASE MSA-batches. Correlation strength was categorized as follows: Excellent (r ≥ 0.95), Very Good (0.85 ≤ r < 0.95), Good (0.70 ≤ r < 0.85), Fair (0.50 ≤ r < 0.70), and Poor (r < 0.50); (b) Distribution of Pearson correlation coefficients of SoP metric and RPS metric across 340 simulated MSA-batches, each reflecting the evolutionary dynamics of an OrthoMaM alignment. (c) Similar to (a), but the metric is TCS score (from reference MSA). (d) Similar to (b), but the metric is TCS score.


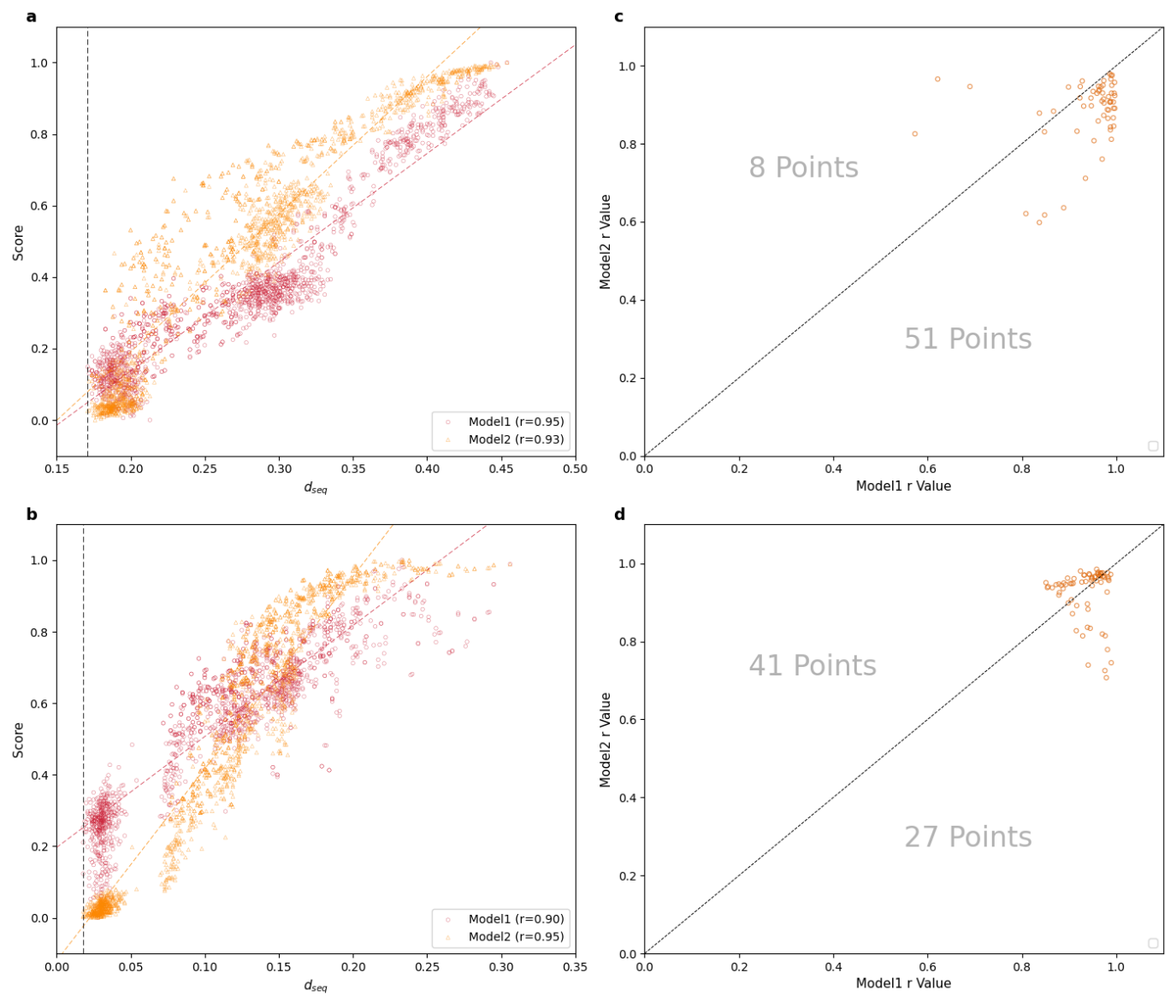


**Supplemental Figure** **S3**. (a) Empirical MSA-batch BBA0169. The Pearson correlation coefficients between an MSA quality score predictors *Model 1* and *Model 2*, and $d_{seq}$ were computed. The quality scores were normalized to be between zero and one, using the formula $\frac{x-min}{max-min}$, where $x$, $min$, and $max$, correspond to the scores of the alternative MSA, the minimal score among all alternative MSAs, and the highest score, respectively. A vertical dashed line indicates the $d_{seq}$ of the most accurate MSA among the alternatives; (b) Similar analysis on a simulated MSA-batch; (c) Distribution of Pearson correlation coefficients across 59 empirical MSA-batches for *Model 1* and *Model 2*; (d) Similar analysis across 68 simulated MSAs.


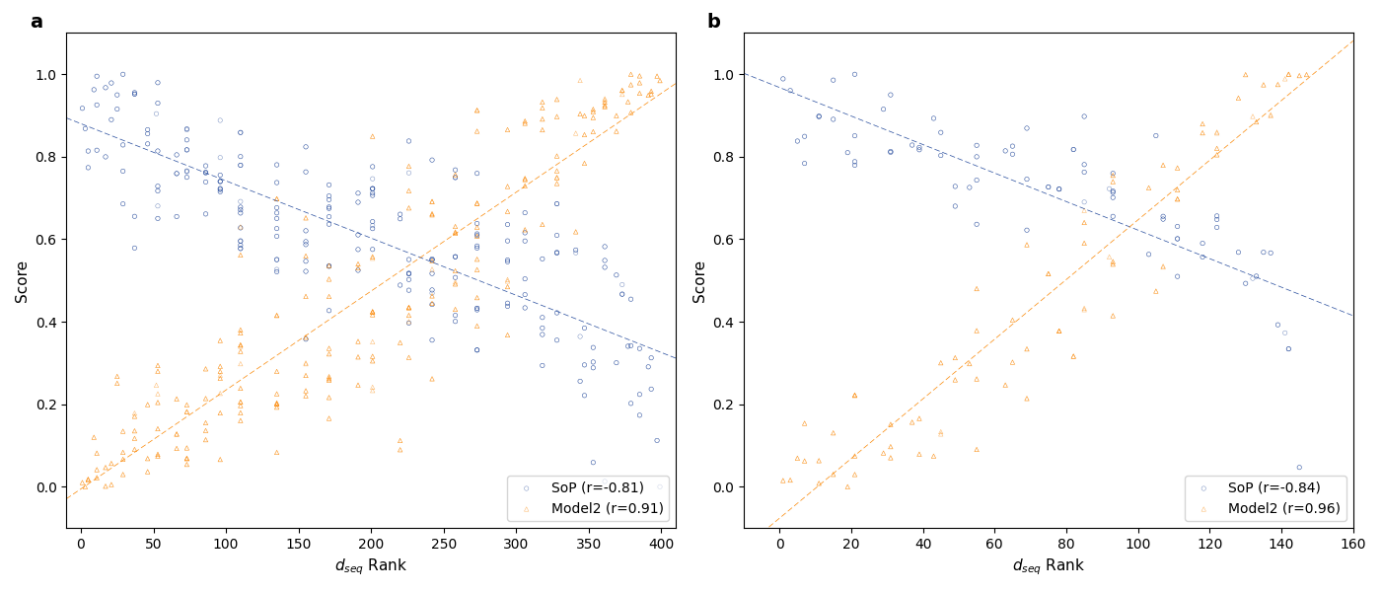


**Supplemental Figure** **S4**. Rank correlations for a simulated protein MSA-batch with (a) 1,000 protein sequences (b) 5,000 protein sequences. The Y axis is the score of either SoP or Model 2, normalized to the range [0,1], as in figure 1 of the main text.


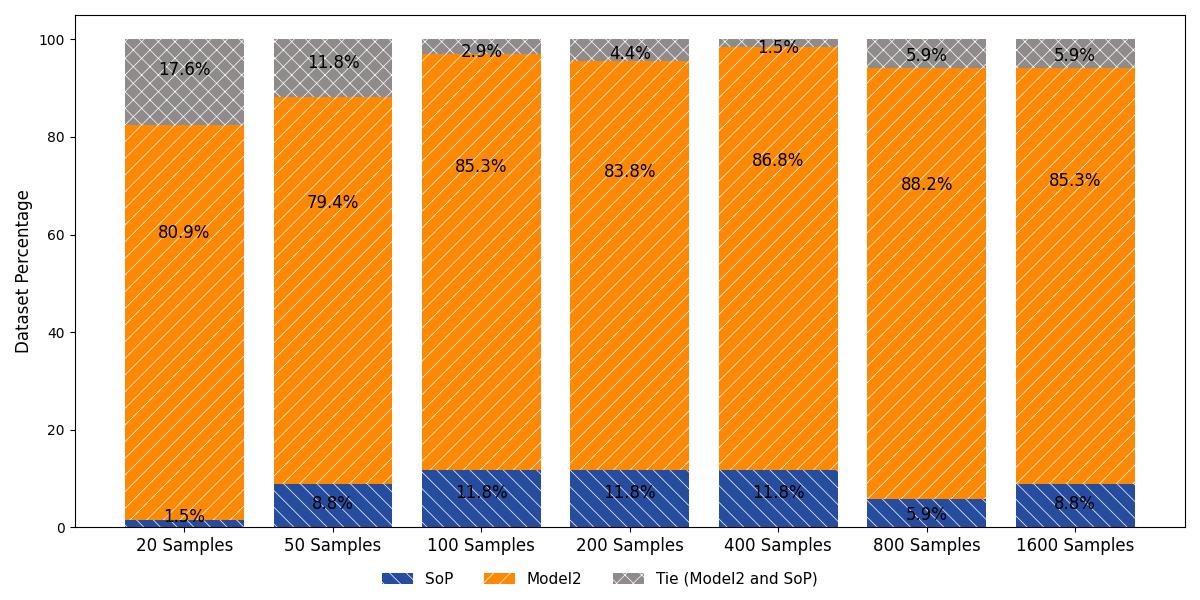


**Supplemental Figure S5**. Performance as a function of the number of alternative MSAs. For each of the 68 held-out test MSAs (i.e., MSA batches not used during training), we randomly sampled 20, 50, 100, 200, 400, 800, or 1,600 alternative MSAs. For each sample size, we report the percentage of cases in which either SoP outperformed Model 2, or vice versa. A tie indicates that both methods selected an MSA with the same distance to the true MSA.


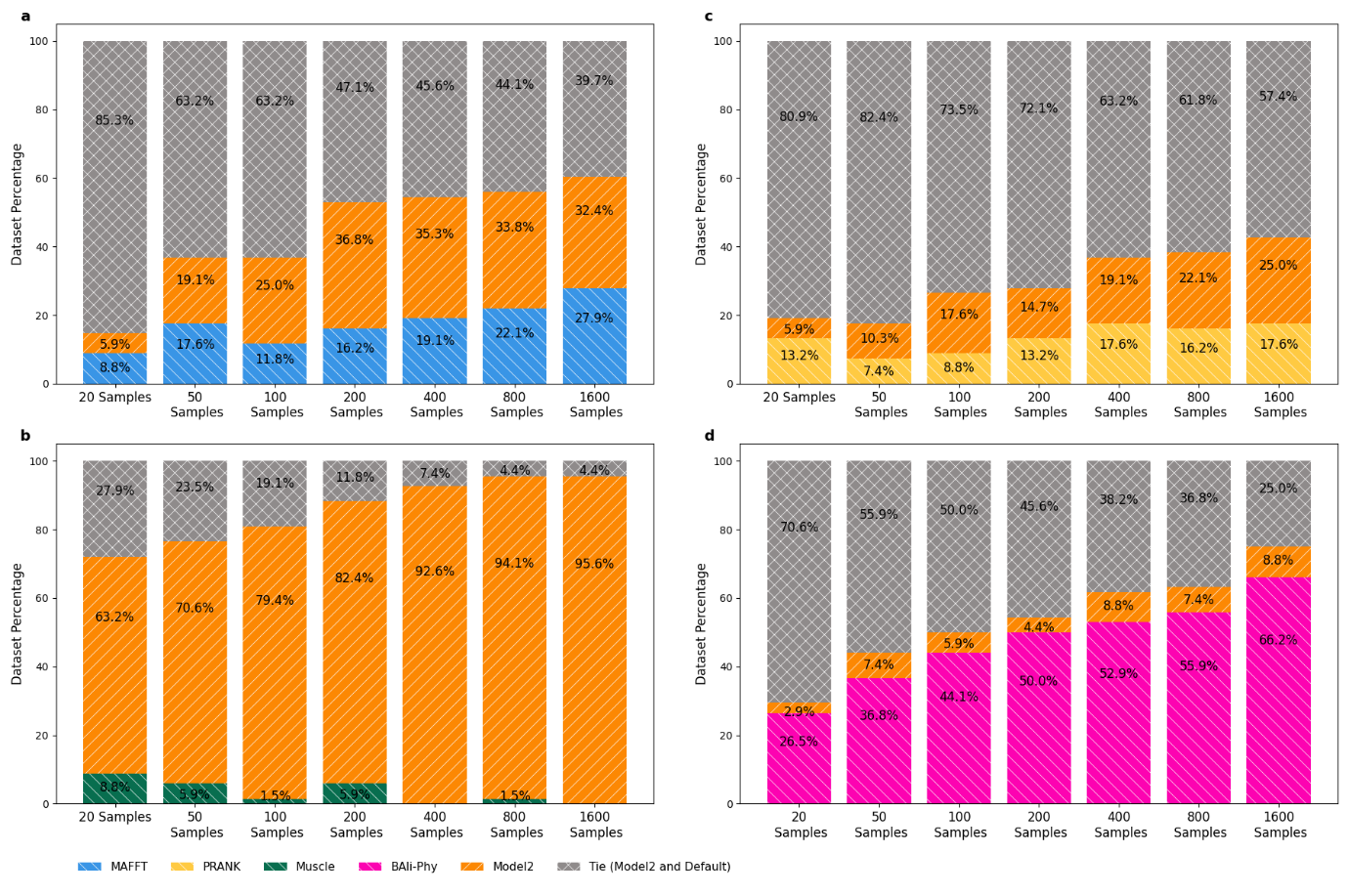


**Supplemental Figure S6**. Performance as a function of the number of alternative MSAs. For each of the 68 held-out test MSAs (i.e., MSA batches not used during training), we randomly sampled 20, 50, 100, 200, 400, 800, or 1,600 alternative MSAs. For each sample size, we report the percentage of cases in which the aligner default outperformed *Model 2*, or vice versa. A tie indicates that both methods selected an MSA with the same distance to the true MSA. (a) Model 2 versus MAFFT; (b) Model 2 versus Muscle; (c) Model 2 versus PRANK; (D) Model 2 versus BALi-Phy.


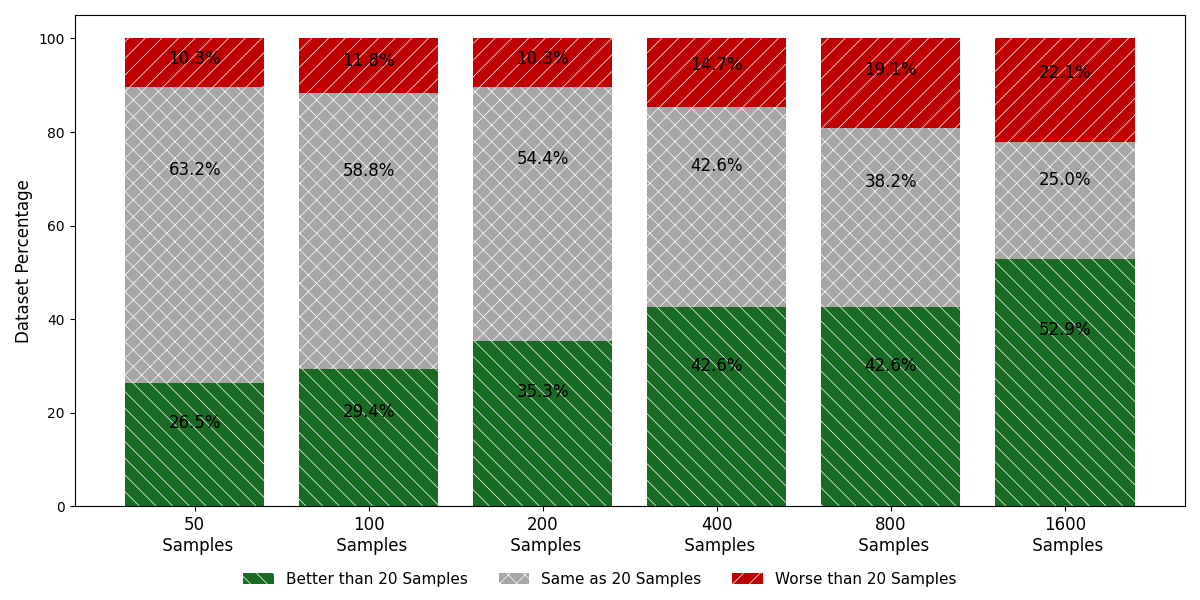


**Supplemental Figure S7**. Performance as a function of the number of alternative MSAs. For each of the 68 held-out test MSAs (i.e., MSA batches not used during training), we randomly sampled 20, 50, 100, 200, 400, 800, or 1,600 alternative MSAs. For sample sizes of 50 or more, we report the percentage of cases in which *Model 2* benefited from the additional alternatives compared to 20 samples, or vice versa. A tie indicates that increasing the number of alternatives above 20 did not result in a more accurate MSA.

**
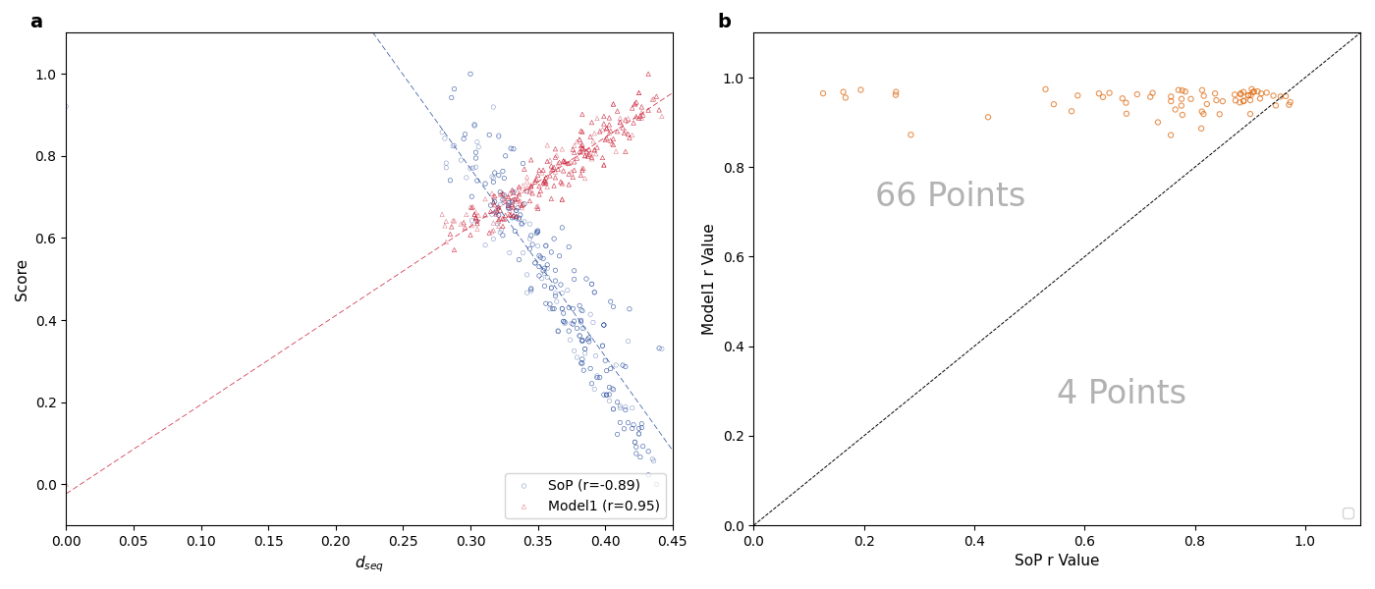
**

**Supplemental Figure** **S8**. (a) The Pearson correlation coefficients between an MSA quality score (either SoP or *Model 1*) and $d_{seq}$ were computed, similar to figure 1A a DNA MSA-batch. A vertical dashed line indicates the $d_{seq}$ value of the most accurate MSA among the alternatives; (b) Distribution of absolute values of Pearson correlation across 70 DNA MSA batches for SoP and *Model 1.* Data-points above the $y=x$ line correspond to cases in which *Model 1* outperformed SoP, i.e., higher correlation coefficients.
